# Management of Gastric Fistulas After Gastric Sleeve Using E-VAC Therapy

**DOI:** 10.3390/diagnostics15212811

**Published:** 2025-11-06

**Authors:** Bogdan Mihnea Ciuntu, Alexandra-Simona Zamfir, Mădălina Maxim, Carmen Lăcrămioara Zamfir, Roxana Elena Ciuntu, Mihai Lucian Zabara, Irina Mihaela Abdulan, Mihaela Corlade-Andrei, Daniel Vasile Timofte, Gheorghe G. Balan

**Affiliations:** 1 Faculty of Medicine, “Grigore T. Popa” University of Medicine and Pharmacy, 700115 Iasi, Romania; bogdan-mihnea.ciuntu@umfiasi.ro (B.M.C.); madalina.maxim@umfiasi.ro (M.M.); mihai-lucian.zabara@umfiasi.ro (M.L.Z.); irina.abdulan@umfiasi.ro (I.M.A.); mihaela.corlade2@umfiasi.ro (M.C.-A.); daniel.timofte@umfiasi.ro (D.V.T.); 2 Department of General Surgery, County Clinical Emergency Hospital St. Spiridon, 700111 Iasi, Romania; 3 Department of Medical Sciences III, Pulmonology, Faculty of Medicine, “Grigore T. Popa” University of Medicine and Pharmacy, 700115 Iasi, Romania; 4 Department of Morpho-Functional Sciences I, Faculty of Medicine, “Grigore T. Popa” University of Medicine and Pharmacy, 700115 Iasi, Romania; carmen.zamfir@umfiasi.ro; 5 Department of Ophtalmology, “Grigore T. Popa” University of Medicine and Pharmacy, 700115 Iasi, Romania; roxana-elena.ciuntu@umfiasi.ro; 6 Emergency “St. Spiridon” County Hospital, 700111 Iasi, Romania; 7 Medical Semiology and Gastroenterology, Department of Medicine I, Faculty of Medicine, “Grigore T. Popa” University of Medicine and Pharmacy, 16 Universitatii Street, 700115 Iasi, Romania; gheorghe-g-balan@umfiasi.ro; 8 2nd Gastroenterology Ward, “St. Spiridon” County Hospital, Independence Bvd. No 1, 700111 Iasi, Romania

**Keywords:** gastric fistula, endoluminal vacuum assisted closure, sleeve gastrectomy, obesity, multidisciplinary approach

## Abstract

**Background and Clinical Significance:** Sleeve gastrectomy is an effective and widely performed bariatric procedure that provides long-term, sustained weight loss, but it carries risks of early and late complications. Among these, gastric fistula is a rare occurrence associated with an increased mortality rate and must be carefully considered to ensure timely diagnosis and appropriate management. **Case Presentation:** We will present the complex case of a patient who was referred to the general surgery department due to severe abdominal pain, exertional dyspnea, nausea, fever and fatigue, symptoms that appeared one month after a robotic gastric sleeve. The investigations led to the diagnostic of high gastric fistula secondary to a gastric sleeve procedure. The patient underwent exploratory laparotomy with jejunostomy, peritoneal lavage, drainage, and endoscopic placement of an endoluminal vacuum assisted closure (E-VAC) system. Close clinical, laboratory, imaging, and endoscopic monitoring demonstrated progressive improvement, with complete resolution of the fistula achieved after seven weeks of E-VAC therapy. **Conclusions:** The particularity of this case lies in the occurrence of a delayed mechanical gastric suture dehiscence, with late diagnosis, managed using E-VAC. Even though rare, gastric fistulas represent a potentially life-threatening complication of sleeve gastrectomy. Early diagnosis and a multidisciplinary approach, which includes infection control, surgical intervention and minimally invasive techniques like E-VAC, are essential for effective management and favorable outcomes.

## 1. Introduction

Obesity, characterized by a body mass index (BMI) of ≥30 kg/m^2^, is a major global public health concern due to its increasing prevalence and association with severe metabolic and cardiovascular complications. While conservative management, including lifestyle changing and pharmacological interventions, remains the primary approach, bariatric surgery has become an essential therapeutic option for patients with severe or refractory obesity. Among the available surgical techniques, sleeve gastrectomy has been widely adopted due to its efficacy and relatively low complication [1].

Long-term studies have demonstrated that, even after a decade, this procedure leads to significant and sustained weight loss, highlighting its critical role in the multidisciplinary and complex management of obesity [2]. Since its introduction, sleeve gastrectomy has gained widespread acceptance and has become a commonly performed bariatric procedure. However, like any surgical intervention, it is associated with a range of potential complications, which can be categorized as early or late, depending on their occurrence before or after 30 days postoperatively. The most frequently reported complications include infection, internal bleeding, abscess formation, staple line leakage and gastric stenosis. In addition to these, rare but severe complications may arise, such as gastric fistula, which can become a life-threatening emergency, particularly when accompanied by hemodynamic instability or sepsis [3,4].

In this context, we present a rare case of gastric fistula following a previous robotic sleeve gastrectomy, emphasizing the diagnostic challenges, clinical course and multidisciplinary approach to its management. Although gastric fistula is an infrequent complication of sleeve gastrectomy, its potential severity requires prompt diagnosis and appropriate treatment.

## 2. Case Report

We present the case of a 50-year-old female patient with a history of grade III obesity and arterial hypertension, who was referred to the general surgery department due to severe abdominal pain, exertional dyspnea, nausea, fever and fatigue. Her medical history indicated that she had undergone a robotic sleeve gastrectomy one month prior presentation. The blood test revealed leucocytosis with lymphocytosis and neutrophilia, as well as lymphocytopenia.

**Figure 1:**
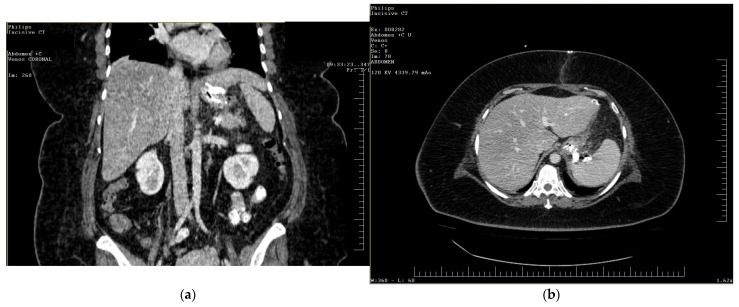
(**a**,**b**) Abdominal computer tomography (coronal and axial view)—parietal defects are observed with the presence of the contrast substance in abdominal cavity.

To gain a more comprehensive understanding of the patient’s condition, a contrast-enhanced abdominopelvic computer tomography (CT) scan was performed. Imaging revealed a reduced gastric volume with the nasogastric tube in place, previously positioned with its distal tip at the subcardial region (Figure 1a,b). In the gastric fornix, two parietal defects were observed, measuring 13 mm (on the posterior wall) and 3 mm (on the anterior wall), with the passage of orally administered contrast substance into the fat of the gastro-splenic ligament. Near the greater curvature of the stomach, a few gas bubbles were present in the omental fat. Further investigations were carried out, including an endoscopic examination, which revealed the esophagus with a 20 mm lumen and tertiary contractions. Additionally, a highly intense pneumatosis was observed throughout the entire colonic frame. The contrast agent administered during the procedure was later detected at the level of the left colon, further guiding the diagnostic process. Following this thorough clinical and paraclinical evaluation, the diagnosis of a high gastric fistula secondary to a gastric sleeve procedure was confirmed.

**Figure 2:**
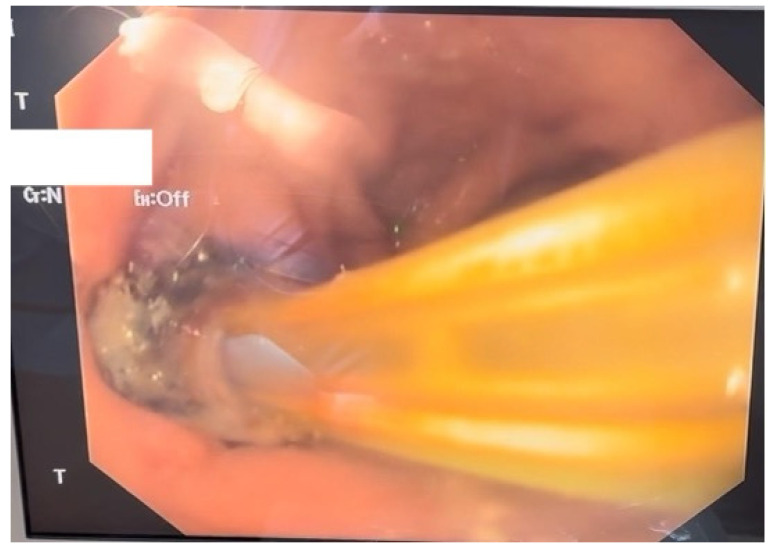
Endoscopic aspect of the E-VAC system inserted through the parietal defect.

Given the severity of the condition, surgical intervention was deemed necessary; therefore the patient underwent an exploratory laparotomy for definitive therapeutic intervention. The procedure involved the creation of a feeding jejunostomy, peritoneal lavage, placement of two drainage tubes and upper gastrointestinal endoscopy with the insertion of an endoluminal vacuum-assisted closure (E-VAC) system (Figure 2).

**Figure 3:**
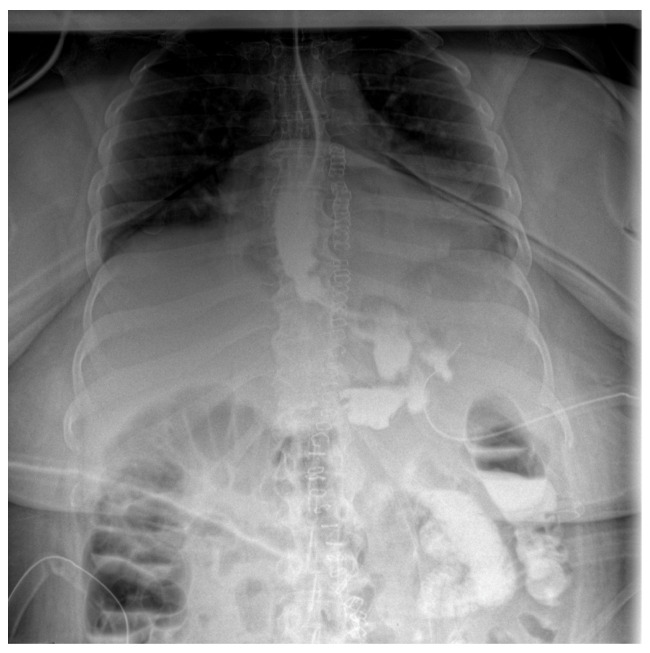
Contrast abdominal radiography—with E-VAC in place without leakage of the contrast substance.

Follow-up staged procedures were performed at 6–7-day intervals, consisting of endoscopic replacement of the E-VAC system at the fistula site, with continuous monitoring of its evolution through direct endoscopic assessment. After six consecutive exchanges of the double-lumen endoluminal vacuum system, endoscopic evaluation confirmed complete closure of the fistulous tract, allowing the gradual reintroduction of oral intake (only liquid form). Under close supervision, the patient tolerated oral feeding for one week, with serial contrast studies using iopamidol confirming the absence of residual fistulous communication (Figure 3).

Throughout the hospitalization, the patient’s recovery progressed steadily, allowing the gradual removal of sutures and drainage tubes. Simultaneously, blood tests demonstrated dynamic changes, reflecting a continuous improvement in clinical status. By the end of the monitoring period, there was a significant reduction in inflammatory markers (leucocytes and C-reactive protein) and hemoglobin values. Hemoglobin remained relatively stable, with only minor fluctuations, while leucocyte counts peaked around the third week before normalizing. CRP levels declined sharply during the first half of hospitalization and stayed low thereafter, consistent with the resolution of the inflammatory process.

**Figure 4:**
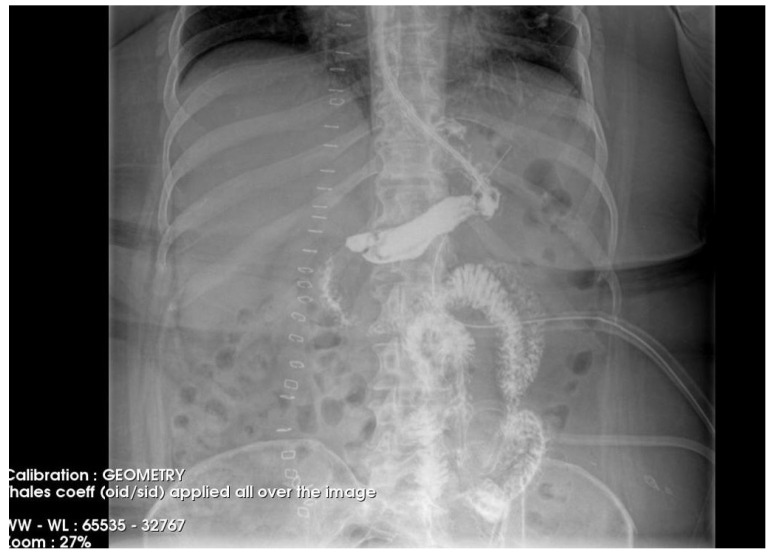
Contrast-enhanced abdominal radiograph demonstrating complete resolution of the fistulous tract, with no contrast leakage or communication visible.

**Figure 5:**
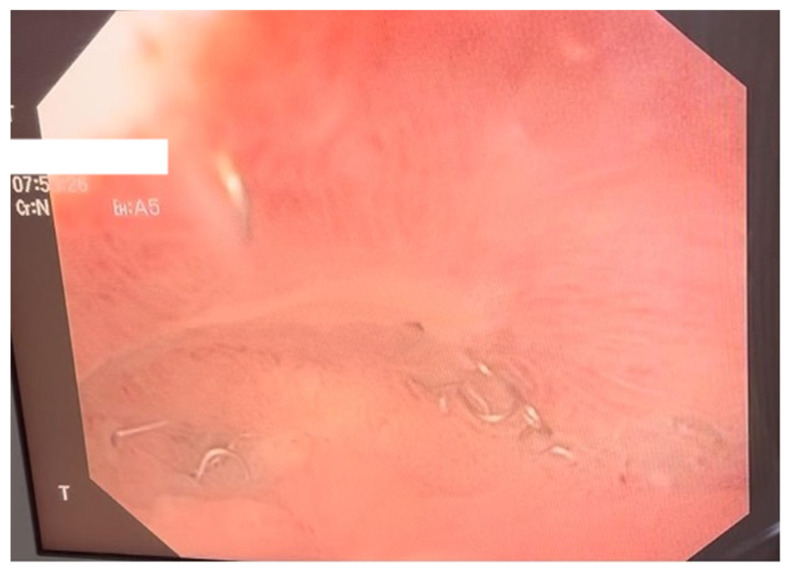
Endoscopic control following vacuum therapy. The image shows the aspect of the defect after completion of therapy, with complete healing of the fistula and visualization of the stapler line after gastrectomy.

The postoperative course remained favorable (Figure 4 and Figure 5), allowing the medical team to discharge the patient after 51 days with specific recommendations.

The patient was advised to adhere to a structured hygienic-dietary regimen, with oral intake initially restricted to 250 mL of liquids per day, while the remaining nutritional requirements were maintained via the jejunostomy. Weekly follow-up consultations were scheduled to monitor progress and determine the appropriate timing for jejunostomy discontinuation. Additionally, an endoscopic reassessment was planned at one month to evaluate gastric healing. Meanwhile, anticoagulant therapy was continued as per protocol to minimize the risk of thromboembolic complications.

## 3. Discussion

Sleeve gastrectomy has been established as an effective and low-risk surgical intervention for the management of class II and III obesity. This procedure involves the resection of the greater curvature of the stomach while preserving the pylorus, resulting in a tubular, reduced-capacity stomach [5]. Moreover, since the duodenum remains intact, the physiological mechanisms responsible for the absorption of essential micronutrients, including vitamins and minerals, remain largely unaffected [6]. As a consequence, patients experience earlier satiety and reduced food intake due to both mechanical restriction and alterations in hormonal regulation [5].

A significant physiological effect of this intervention is the decreased secretion of ghrelin, a peptide hormone predominantly produced in the gastric fundus, which plays an essential role in appetite stimulation and glycemic regulation. Under normal physiological conditions, ghrelin levels achieve the highest value preprandially, which starts to decline approximately one hour postprandially. By diminishing ghrelin production, sleeve gastrectomy contributes to appetite suppression, improved metabolic control, and sustained weight loss outcomes [5,7].

As with any surgical procedure, sleeve gastrectomy is associated with potential complications, which are classified as early, if they occur within the first month postoperatively, and late, if they arise thereafter. Among early complications, the most prevalent is internal bleeding, reported in up to 5% of cases. Another significant early complication is anastomotic leakage, most commonly occurring beneath the gastroesophageal junction [8]. Partial splenic ischemia may occur when arterial supply is compromised due to the division of fundus attachments. Regarding late complications, the most common include gastric stenosis, gastroesophageal reflux disease or the depletion of essential nutrients [3,6].

Rare complications such as gastric fistulas, which are associated with a mortality rate of up to 9%, must be carefully considered to ensure timely diagnosis and appropriate management. It is very important to highlight that, to date, no standardized guidelines have been developed for the management of this complication [4]. Another point of discussion concerns the correlation between the timing of fistula onset and its severity. Existing studies indicate that neither early nor late presentation serve as reliable prognostic factors for patient outcomes [8]. In the present case, the fistula was diagnosed approximately one month after the initial sleeve gastrectomy.

The diagnosis is typically established through a comprehensive anamnesis, where patients report a history of sleeve gastrectomy, along with clinical manifestations indicative of an acute syndrome, primarily characterized by abdominal pain. Additionally, non-specific signs suggestive of infection may be present, as evidenced by elevated inflammatory markers in blood tests. To confirm the diagnosis, imaging studies play a crucial role. Contrast-enhanced CT is recommended to assess the presence, location and extent of the complication [8]. It is equally essential to identify the underlying cause of fistula formation in order to guide appropriate management and prevent recurrence. Furthermore, it is necessary to determine the exact anatomical nature of the fistula—whether gastric, gastropleural, or gastropulmonary—as these last variants can occur following sleeve gastrectomy and are frequently associated with significant respiratory complications [9,10]. Early and accurate classification not only influences therapeutic decision-making, but also highlights the importance of multidisciplinary approach.

Highly digestive fistulas, especially those that occur after bariatric procedures such as sleeve gastrectomy or gastric bypass, represent a severe complication associated with high morbidity and an increased risk of sepsis. In recent years, endoscopic treatment has become the essential component of the management of these lesions, with the main options being self-expanding metal stents (SEMSs) and endoluminal negative pressure therapy (Endoscopic Vacuum Therapy—EVT) [11].

SEMS acts by excluding the fistulous defect from the digestive circuit, ensuring a diversion of enteral flow and mechanical protection of the dehiscence area. In contrast, EVT is based on the application of controlled negative pressure (−75 to −125 mmHg) for the drainage of the fistulous cavity, local decontamination and stimulation of tissue granulation. Thus, while the stent provides passive isolation, EVT actively promotes biological healing of the defect. The indications of the two methods differ depending on the morphology and evolution of the fistula. SEMS is indicated in small, recent fistulas without associated collection, while EVT is preferred in the presence of peri-anastomotic cavities, septic collections, or chronic fistulas. In these situations, active drainage and stimulation of tissue regeneration make EVT a superior alternative [12,13,14].

Several comparative studies support the superior efficacy of EVT. Mandarino et al. (2023) reported, in a meta-analysis of 357 patients, a significantly higher overall closure rate for EVT compared to SEMS (odds ratio 2.58; 95% CI 1.43–4.66), as well as a lower mortality in the vacuum-treated group [12]. A meta-analysis dedicated exclusively to post-bariatric fistulas revealed a clinical success rate of approximately 87.2% for EVT [13]. Recent studies in series of patients undergoing esophagogastric or bariatric surgery have confirmed similar results, with healing rates of over 90% and a favorable safety profile [14,15].

The mean duration of treatment is comparable between the two methods (4–6 weeks), but EVT generally requires a change of system every 3–5 days. The main complications of SEMS are migration (up to 30%), pain and mucosal erosions, while EVT is associated with only minor discomfort and rarely bleeding [12,14].

In the context of bariatric surgery, EVT has established itself as the method of first choice in fistulas with cavities or local sepsis, offering superior infection control and faster tissue healing. SEMS remains useful in simple fistulas without collection, or in centers without experience in EVT. The combined approach, which associates endovacuum drainage with the placement of a protective stent, offers promising results in complex cases [15].

According to the recent guidelines of the European Society of Gastrointestinal Endoscopy (ESGE, 2023–2025), EVT is recommended as a first-line strategy for complicated digestive fistulas, while SEMS can be reserved for simple lesions or situations where vacuum therapy is not available [12,14]. In conclusion, the choice of endoscopic method should be adapted to the size and complexity of the defect, the presence of collections and the available resources. SEMS offers an effective solution for isolated fistulas, while EVT has been shown to be superior in complex or septic fistulas, ensuring a higher healing rate and reduced morbidity.

Furthermore, endoscopy serves as a valuable diagnostic tool, enabling direct visualization of the fistula, thereby facilitating appropriate treatment planning [8]. In this case, the patient had previously undergone a laparoscopic sleeve gastrectomy abroad, which subsequently led to the development of an abscess, followed by a fistula. Given the presence of the symptomatology suggestive for an infection, prompt initiation of broad-spectrum antibiotic therapy was warranted, in order to control the systemic infection and mitigate the risk of sepsis. Afterwards, surgical intervention was performed, with laparotomy being preferred as it allowed multiple procedures to be performed during the same intervention (multiple drainage of the abdominal cavity, finding and placing a drainage tube near the fistula and creating a feeding jejunostomy). Intraoperatively, a feeding jejunostomy was established to ensure adequate enteral nutrition, followed by the peritoneal lavage. To facilitate continuous drainage and prevent the accumulation of intra-abdominal fluid collections, two drainage tubes were inserted. Additionally, upper gastrointestinal endoscopy was conducted to assess the fistulous tract and guide further management. As part of a minimally invasive strategy, an E-VAC system was deployed to promote wound healing by facilitating continuous drainage, reducing local contamination, as well as enhancing tissue granulation. Previous studies have demonstrated favorable outcomes with E-VAC therapy, particularly in patients with upper gastrointestinal involvement [16].

Throughout the hospitalization, the patient underwent continuous hemodynamic monitoring, including serial blood tests to assess inflammatory markers and overall systemic response. Imaging studies, particularly CT, were regularly performed to evaluate the progression of the fistula, detect any potential complications, and guide further therapeutic decisions. At the same time, endoscopic assessments were conducted to directly visualize the fistula and monitor its evolution under E-VAC therapy. The resolution of the fistula was observed after the seventh week of treatment, suggesting a gradual healing process.

Endoluminal negative pressure therapy (ENPT) has been introduced as an effective therapeutic modality for the management of postoperative fistulas of the upper gastrointestinal (GI) tract, building upon principles initially applied in the treatment of rectal fistulas, as first described by Weidenhagen et al. and subsequently adapted for upper GI pathology by Wedemeyer et al. [17,18]. In recent years, several comparative studies have evaluated the efficacy of ENPT against self-expandable metallic stents (SEMSs) for this indication, with results generally favoring ENPT in terms of clinical outcomes and complication rates [19,20,21].

In the analyzed clinical cases, a sponge sleeve—through which a double-lumen suction tube was passed—was anchored at both the proximal and distal ends using 2/0 Vicryl sutures. This specific technique, developed through iterative clinical adjustments, significantly improved the mechanical stability of the assembly. The ends of the sponge were fixed to the aspiration tubing by suturing directly through the tubing wall, ensuring that the suction surface remained unobstructed. This approach not only prevented the sponge from migrating along the tube’s length, but also considerably reduced the risk of system rupture during removal. Furthermore, the anchoring method enhanced the endoscopist’s ability to control the device’s positioning at the site of the defect, an essential factor considering the limited space for maneuvering adjacent to the endoscope during insertion.

While the use of endoscopic clips is typically limited to defects <25 mm and SEMS placement carries significant risks (such as stent migration, patient discomfort, bleeding, and tissue ingrowth through the stent mesh, leading to several complications and subsequent removal), ENPT use brings several advantages. These include the ability to custom-shape the sponge to the size and contour of the defect and to perform active, continuous drainage of the entire fistulous cavity. Pre-procedural planning for ENPT requires accurate estimation of the defect’s dimensions, typically achieved through endoscopic visualization and CT imaging. These parameters are critical in selecting an appropriately sized foam sponge and the nasogastric or drainage tube onto which the sponge will be mounted.

Regarding the optimal frequency of sponge exchange, the literature describes different replacement intervals ranging from every 2–4 days to every 7 days [22,23,24]. In our experience, we observed a consistent increase in inflammatory markers after the fourth day of continuous therapy, despite performing two lavage cycles per day with antiseptic and antibiotic solutions tailored according to antibiogram (in instillation volumes not exceeding 40 mL per cycle). Extending the interval between instillations, however, appeared to delay the inflammatory response and, in some cases, allowed the sponge to remain in place for over five days without clinical deterioration.

These findings are consistent with other reports, which document favorable outcomes even when sponge replacements are performed every 7 to 14 days [25]. These results have been further reinforced by updates to ENPT protocols since 2018, recommending weekly sponge changes. Nonetheless, the frequent replacement of the vacuum system increases treatment costs and exposes the patient to multiple endoscopic procedures under general anesthesia—interventions that may not yield proportional clinical benefit. At the same time, excessively prolonged retention of the system can result in firm adhesion of the sponge to the wound bed, thereby complicating removal and making it more difficult to monitor the evolution of the defect.

In our case, we performed a thorough endoscopic and radiologic assessment of the defect and the surrounding cavity together with continuous monitoring of inflammatory and clinical parameters, which allowed us to individualize the timing of sponge exchanges. Lavage with antiseptic solutions—such as povidone-iodine or saline—proved effective for clearing the sponge; we observed a marked increase in fluid collected in the vacuum canister shortly after these procedures. The microbiological and macroscopic analysis of the aspirated material frequently revealed small fragments of necrotic tissue, indicating the system’s efficacy in debriding and evacuating nonviable tissue [26].

In our case, we temporarily reduced the negative pressure setting to 40 mmHg during instillation, which facilitated detachment of necrotic material from the sponge’s surface by creating a cleavage plane. Upon the reactivation of the vacuum system, the instilled fluid and dislodged debris were rapidly aspirated and collected in the drainage reservoir. The entire instillation–evacuation cycle typically lasted less than 10 min. It is important to note that if instillation is performed without temporarily disabling or reducing suction, the fluid tends to be aspirated immediately through the path of least resistance, diminishing its therapeutic effect.

Negative pressure wound therapy with instillation (NPWTi) facilitates the consistent irrigation of the wound, thus enhancing therapeutic outcomes, although it also increases the procedural complexity. NPWTi is especially valuable in patients with high bacterial load or multiple comorbidities, as it allows for decontamination and removal of infectious or necrotic material [27]. The clinical benefits of NPWTi in complex wounds arise from several physiological mechanisms: reduction in bacterial burden, dilution of inflammatory and cytotoxic mediators, improved tissue hydration, and stimulation of angiogenesis through intermittent vacuum cycling [28]. While conventional NPWT is known to aid in exudate and bacterial removal, its efficacy in significantly reducing the microbial burden still remains uncertain [29,30,31]. Nevertheless, irrigation remains a central element in the management of contaminated wounds, facilitating uniform distribution of antiseptic or antibiotic agents across the entire surface, including in poorly accessible areas [32].

Experimental research has shown that NPWTi outperforms low-pressure irrigation systems in removing wound debris and significantly reduces cross-contamination and aerosolization of bacteria [33]. Even the use of saline alone has been shown to aid in the mechanical removal of microbial colonies from both the wound bed and the sponge. Additionally, micro-deformations generated during vacuum cycling contribute to the movement of sponge pores relative to the tissue, disrupting biofilm formation and facilitating necrotic tissue detachment.

In our experience, the double-lumen suction tube used in ENPT offered an additional clinical advantage: the ability to verify cavity sealing following instillation. This was achieved by connecting a syringe to the secondary lumen during vacuum reactivation; the free movement of the syringe plunger confirmed the effective retrograde transmission of negative pressure and, therefore, the integrity of the closed system. The presence of a guidewire and calibration markings on the tube further allowed us to monitor its position precisely throughout therapy. This configuration also enabled us to deliver antiseptic or antibiotic solutions directly to the wound site and immediately reaspirate them. Aside from controlling local inflammation, this lavage technique also facilitated the easier detachment of the sponge from granulating tissue during system replacement. Pre-procedural instillation of 20–30 mL saline and stopping the vacuum 30 min before the endoscopic intervention proved useful in shortening procedure time and minimizing bleeding risk during mobilization of the distal end of the tube.

The main therapeutic advantage of ENPT lies in its ability to evacuate fluid collections and promote healing in anatomically complex and surgically inaccessible cavities, using a minimally invasive approach. This method may help prevent systemic complications such as sepsis, thereby improving patient outcomes and reducing mortality [34]. Although treatment duration may be extended, ENPT is supported by strong clinical success rates when compared to the high mortality associated with non-treated or conventionally managed fistulas [35].

The management of the upper GI tract perforations requires close clinical monitoring, intensive care support, and individualized surgical decision-making, based on factors such as hemodynamic stability, the extent of contamination, underlying comorbidities and perforation site. Optimal outcomes are achieved through a multidisciplinary approach involving surgeons, endoscopists, radiologists, internists and critical care specialists. Although ENPT is minimally invasive in principle, it may necessitate adjunctive surgical procedures, such as thoracotomy with mediastinal drainage or the placement of a feeding jejunostomy. Ultimately, integrated medical and surgical care is pivotal for therapeutic success. As a relatively novel technique, ENPT represents a promising and increasingly validated alternative for the management of complex upper gastrointestinal perforations.

## 4. Conclusions

The management of upper gastrointestinal tract fistulas requires a multidisciplinary approach, with therapeutic decisions guided by hemodynamic stability, the degree of contamination, and patient-specific factors.

Endoluminal negative pressure therapy (EVT) has established itself as the modern standard in the management of post-bariatric high digestive fistulas, demonstrating superior fistula closure rates and effective control of local sepsis compared to traditional methods, such as self-expanding metal stents. By actively draining the peri-anastomotic cavities and stimulating tissue regeneration, EVT accelerates healing and reduces the risk of complications, while offering a favorable safety profile. The flexibility of the method allows adaptation to various types of defects and combination with other endoscopic techniques, which justifies the recommendation of European specialist guidelines to consider it as first-line therapy in centers with advanced experience in post-bariatric endoscopy.

## Data Availability

The data published in this research are available on request from the first author and corresponding authors.
